# Assessment of usefulness of synchrotron radiation techniques to determine arsenic species in hair and rice grain samples

**DOI:** 10.17179/excli2016-785

**Published:** 2017-01-02

**Authors:** Jun Lin, Guo-fang Lin, Yu-lan Li, Xiao-yan Gao, Hui Du, Chao-gang Jia, Hong-chao Lu, Klaus Golka, Jian-hua Shen

**Affiliations:** 1 Shanghai Institutes for Biological Sciences, Institute of Plant Physiology and Ecology, Chinese Academy of Sciences, Shanghai 200032, China; 2 Shanghai Institute of Applied Physics, Chinese Academy of Sciences, Shanghai 201800, China; 3 Prefecture Center of Disease Prevention and Control of Southwest Guizhou Ethnic Bouyei and Hmong Autonomous Prefecture, Xingyi, Guizhou 562400, China; 4 County Institute of Public Health Supervision of Xingren, Xingren, Guizhou 562300, China; 5 Leibniz-Institut für Arbeitsforschung an der TU Dortmund - Leibniz Research Centre for Working Environment and Human Factors, 44139 Dortmund, Germany

**Keywords:** arsenic, speciation, synchrotron radiation, biogeochemical abnormality, arseniasis, micro-distribution

## Abstract

The arseniasis in Southwest Guizhou, China has been identified as a unique case of endemic arseniasis caused by exposure to indoor combustion of high As-content coal. Present investigation targeted the microdistribution and speciation of the element arsenic in human hair and environmental samples collected in one of the hyper-endemic villages of arseniasis in the area. Analyses were performed by micro-beam X-ray fluorescence (μ-XRF) and X-ray absorption fine structure (XAFS). The total As level in hair samples of diagnosed patients was detected at almost the same level as in their asymptomatic neighbors. Concentrations in the lateral cut of hair samples were high-low-high (from surface to center). XAFS revealed the coexistence of both the As^+3^ and As^+^5 states in hair samples. However, the samples from patients displayed a tendency of higher As^+3^ / As^+5^ ratio than the asymptomatic fellow villagers. The μ-XRF mapping of rice grains shows that arsenic penetrates the endosperm, the major edible part of the grain, when rice grains were stored over the open fire of high As-content coal. Synchrotron radiation techniques are suitable to determine arsenic species concentrations in different parts of hair and rice grain samples. As arsenic penetrates the endosperm, rinsing the rice grains with water will remain largely ineffective.

## Introduction

Due to various geological mechanisms or anthropogenic activities, an altered distribution of certain chemical elements occurs on the surface in some parts of the earth. The over- or under-abundance of certain chemical elements will result in an imbalance of element exchange between human bodies and the environment. If the exchange exceeds the normal range that the organism is able to buffer or to tolerate, certain kinds of health disorders will emerge. The major biogeochemical abnormality-related endemics known so far in China include endemic arseniasis (which covers two kinds of As exposure routes: one via As polluted drinking water sources and another one via indoor combustion of high As-content coal), fluorosis (via polluted water or via indoor air pollution from high fluorine-content coal burning and from brick tea), iodine deficiency disorders (IDD), and other diseases. 

Several villages in Southwest Guizhou Bouyei (Buyi) and Hmong (Miao) Ethnic Minority Autonomous Prefecture, Southwest China represent a unique case of endemic arseniasis, which is related to indoor combustion of high arsenic-content coal, not with As polluted drinking water sources (Jin et al., 2003[7]; Zheng et al., 2005[20]; Liu et al., 2002[13]).

Since the early 1960s, as local woods and bushes have been depleted, villagers in the above mentioned area burned local high As containing coal in poorly or unventilated stoves (without chimneys) for cooking, heating and drying crop and food. The highest As concentration in local coal was once detected as 3.2-3.5 % (Ding et al., 2001[2]). Since the early 1970s thousands of arseniasis cases emerged. The area where the target village of the present investigation is located was the first one reported (Zhou et al., 1993[21]). Most of the cases diagnosed by the end of the millennium (1,386 out of 2,241 cases) are clustered in the township where the target village of the present investigation is located (Jin et al., 2003[7]).

Most of the work about this endemic population reported so far was focused on environmental causes and confirmed the causality between indoor burning of high arsenic-content coal and the excess prevalence of arseniasis cases in rural population (Jin et al., 2003[7]; Zheng et al., 2005[20]; Liu et al., 2002[13]; Zhou et al., 1993[21]). Variation in individual susceptibility to the chronic poisoning of inorganic arsenic exposure has been suggested (Vahter, 2000[16]). In our early field survey conducted in 2002, a remarkable ethnicity-dependent (or clan-dependent) difference of arseniasis prevalence has been observed in the residents with different ethnic origin and various clan relationships in the same village. This was surprising as the families of different ethnicity and of different clans have been living together in the same village for generations (Lin et al., 2003[8]) and were exposed likewise to indoor combustion of local high As coal for quite similar time duration (Lin et al., 2006[10]). An array of host factors has been proved to be related to the different impact of susceptibility to arseniasis risk under this unique As exposure scenario (Lin et al., 2006[10], 2007[11], 2010[12][9]).

New techniques for the *in situ *detection of micro-distribution and speciation of the element arsenic in specimens of exposed persons and in polluted environment are required for further expanding our knowledge of various risk factors. This may pave the way for a quantitative understanding of all the factors which might influence the excess risk of arseniasis, irrespective of whether or not exposure-related, host-related, or related to a combination of both. The utilizing of synchrotron radiation techniques, such as micro-beam X-ray fluorescence (μ-XRF) and X-ray absorption fine structure (XAFS), might be a promising option (Gault et al., 2008[5]). Both, μ-XRF and XAFS are non-destructive physical procedures and have been widely used to detect, quantify and map the element content and the speciation of samples in their natural state. In this work, distribution and speciation of the element arsenic within a single human hair or rice grain was determined using the well-established μ-XRF mapping procedure and XAFS technique in Shanghai Synchrotron Radiation Facility (SSRF, Shanghai, China).

## Material and Methods

### Sample collection

Human hair samples as well as rice and corn samples were collected in one of the hyper-arseniasis-endemic villages exposed to indoor combustion of local high-As content coals for decades. 

A cross-sectional epidemiologic field study was conducted on all members of both clans in the target village in April 2004. Only the members related by blood and their spouses were included. The arseniasis cases in both ethnic clans were diagnosed according to “Diagnosis guideline for arseniasis, WS/T 211-01” issued by the Chinese State Ministry of Health. On the basis of the epidemiologic field study an Excel^TM^-based database was created. The samples for this research project were collected from the target village considering the representation of the population investigated (ethnicity, lineage, gender, diagnosed disease, etc.).

The hair samples were donated by diagnosed arseniasis patients as well as by their asymptomatic fellow villagers. All the participants gave their formal consent. The participants included: 10 diagnosed arseniasis patients (7 males and 3 females) 47.6±13.0 years old (mean±SD) and 6 arseniasis-asymptomatic fellow villagers (3 males and 3 females) 49.5±17.4 years old. The rice and the corn samples which were kept over the open fire of high As-content coal for drying were collected in the farmer families in the same endemic village. The control samples of rice and corn (which were never baked over an open fire of high As coal) were taken from a non-arseniasis endemic township in the same county where the As concentration of the coal farmers there used for domestic purposes proved to be within the normal range. Hair samples were also collected from a unique thallotoxicosis-endemic village in the same county (n=6, incl. 3 males and 3 females; thereof 3 diagnosed thallotoxicosis patients and 3 thallotoxicosis-asymptomatic individuals, 49.5±17.4 years old). In this village the only case worldwide of thallotoxicosis caused by natural exposure (the biogeochemical abnormality of the element thallium in soils near the village), instead of pure and direct anthropogenic reasons, such as poisoning or accident has been reported (Xiao et al., 2004[19]). The exposure route for thallium was eating crops grown on thallium enriched soil. There is no statistically significant age difference among all subgroups of rural residents (P=0.846). 

### Synchrotron radiation micro-beam X-ray fluorescence spectrometry (μ-XRF) and X-ray absorption fine structure (XAFS) analysis

μ-XRF experiments were carried out on the BL15U beamline station at the SSRF (Shanghai Synchrotron Radiation Facility). Monochromatic light was obtained using a Si (111) double crystal and then focused to a specified beam size using a K-B mirror. A silicon drift detector (SDD) was used to record the characteristic fluorescence spectra of elements in the samples. Samples were mounted on a stage, which can drive the sample step by step with a step resolution of 3 μm.

The X-ray absorption fine structure spectra were carried out on the BL14W station at the SSRF. Monochromatic light was obtained using a Si (111) double crystal monochromator, with a scanning energy step of 0.5 eV. The XAFS spectra were recorded with fluorescence mode using 4-elements SDD. A filter was placed between the detector and the sample to suppress light scattering.

### Inductively coupled plasma mass spectrometry (ICP-MS) analysis 

Human hair samples were firstly cut into 1 cm long fragments and rinsed with ethanol twice. Samples were dried at room temperature, with a relative humidity of about 30 %. The sample digestion procedure was based on the method published by Uchino et al. (2006[15]) with minor modification: samples were loaded into a Teflon digestion vessel with 3 ml of 35 % nitric acid and 1 ml of hydrogen peroxide. The digestion vessels were then placed in a high-pressure microwave (Ethos 320; Milestone, Italy). A four-stage temperature program with a maximum temperature of 180 °C and a total digestion time of 31 min was used. 

The total As measurements were carried out on an X series 7 ICP-MS instrument (Thermo Scientific, USA) equipped with a concentric nebulizer and hexapole collision cell technique (CCT). ICP-MS operation conditions were as follows: radiation frequency (RF) power was set to 1350 W, carrier gas flow and peristaltic pump rates were 1 ml x min^-1^ and 25 rpm, respectively. The dwell time was set to 10 ms for assay quality control. To validate the measurement of As in hair samples, human hair master standard GBW9101b was employed as certified standard reference material (SRM) for quality assurance.

### Statistical analysis 

To analyze differences between diagnosed arseniasis patients (reference), asymptomatic fellow villagers, and residents of an endemic village of chronic thallotoxicosis, an ANOVA analysis (STATISTICA 6.0, StatSoft Inc.) was performed on the data of As_75_ content of ICP-MS and on the ratio of As^+3^/As^+5 ^data of XAFS as well. P values <0.05 were regarded as significant.

## Results

### Element distribution along the human hair

**Figure 1:**
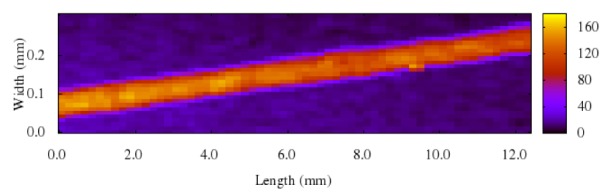
Arsenic distribution in a typical human hair sample of a diagnosed arseniasis patient. Longitudinal cut, length 12 mm.

The arsenic distribution along the hair samples from diagnosed arseniasis patients were mapped along the axial direction. A part of the spectrum is displayed in Figure 1(Fig. 1). The distribution of arsenic in the hair is largely homogenous. It indicates the exposure environment in this village prior to sampling was, basically, stable. 

### Arsenic distribution and species in hair of symptomatic patients

**Figure 2:**
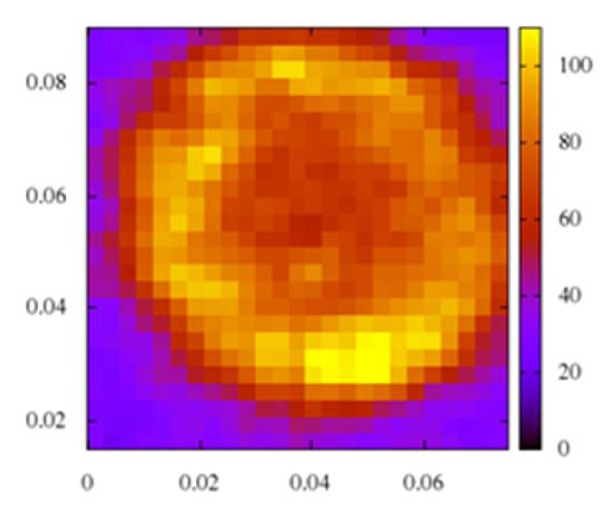
Distribution of As in a hair sample of a diagnosed arseniasis patient by X-ray absorption fine structure (XAFS). Cross section.

The arsenic distribution in hair from diagnosed patients was studied by synchrotron radiation micro-beam X-ray fluorescence spectrometry (μ-XRF) mapping and is presented in Figure 2(Fig. 2). A tendency of “high-low-high” pattern (from the surface to the center) was observed. The spectrum shows that As was concentrated at the surface and more pronounced at the core (medulla of the hair). 

**Figure 3:**
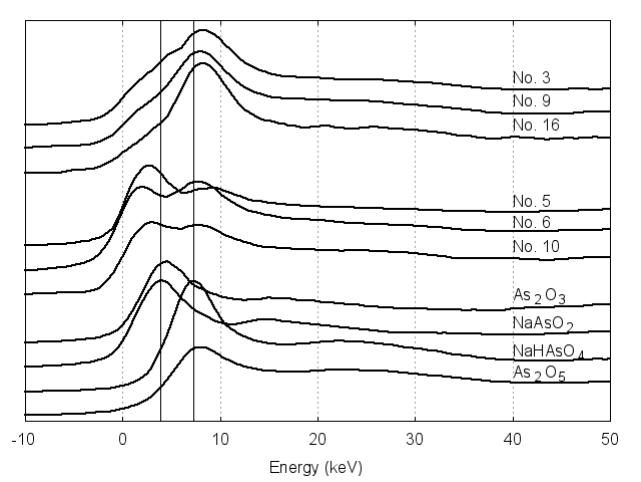
Arsenic speciation in hair samples of residents in an arseniasis hyper-endemic village. Left vertical line: Energy for As^3+^, right vertical line: Energy for As^5+^. As_2_O_5_, NaHAsO_4_: References for As^5+^; NaAsO_2_, As_2_O_3_: Reference for As^3+^.

The XAFS spectra of human hair collected from an arseniasis hyper-endemic village as well as four reference chemicals with different chemical valences of arsenic are displayed in Figure 3(Fig. 3). The samples 5, 6, and 10 are from diagnosed arseniasis patients while samples 3, 9, and 16 represent arseniasis-asymptomatic individuals from the same endemic village. The dominant As speciation displayed among the patients, mostly, was As^+3^, while among the asymptomatic fellow villagers, on the opposite, dominated As^+5^. From the toxicological point of view, As^+3^ compounds are more toxic than As^+5^(Hirano et al., 2003[6]; Styblo et al., 2000[14]).

### The ratio of As^+3^/As^+5^ in human hair samples 

The XAFS data of hair samples from all the individuals, both arseniasis patients and their asymptomatic neighbors are displayed in Table 1(Tab. 1). The data show the ratio in ethnic Hmong individuals was slightly higher than in their ethnic Han neighbors (0.90±0.45 *vs.* 0.77±0.68, F=0.092, p=0.767). Patients show a higher dominance of As^+3^ state over the As^+5^ (0.94±0.66 *vs.* 0.58±0.54, F=1.037, p=0.330). If comparison is made among the ethnic Han villagers, the most arseniasis-susceptible ethnic groups in this area (Lin et al., 2006[10]; Chen et al., 2009[1]), the deviation further increases, though the statistical significance still is not reached (0.99±0.69* vs.* 0.26±0.26, F=2.995, p=0.122).

### ICP-MS comparison of As content in hair samples from arseniasis patients and from asymptomatic individuals 

Table 2(Tab. 2) displays the ICP-MS data of As_75 _in the hair samples of villagers, both diagnosed patients and asymptomatic individuals living in the same hyper-endemic villages. The As_75 _levels were almost equal in the two different groups of the villagers (F=0.001; P=0.981). A comparison was made with a group of farmers of a chronic thallotoxicosis-endemic village (caused by a rare kind of biogeochemical abnormality of the element thallium) in the same county (Xiao et al., 2004[19]). The specimens from thallotoxicosis-endemic villagers show the same arsenic level as in arseniasis-endemic villagers in the same county (F=0.018; P= 0.896). It is worthwhile to note that no typical arseniasis symptoms, e.g. dermatological symptoms, have been diagnosed so far, although the residents in that thallotoxicosis-endemic village have been exposed, at the same time, to very high levels of As and unusual body burdens of As were recorded, too.

### Arsenic distribution in rice grain

**Figure 4:**
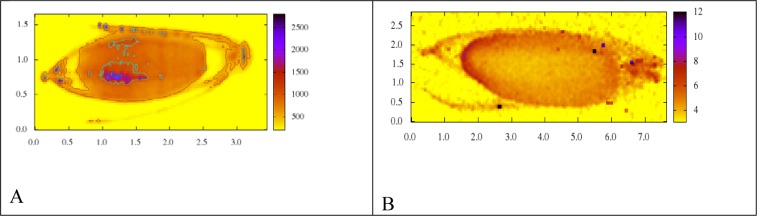
Micro-beam X-ray fluorescence spectrometry (μ-XRF) mapping of a rice grain A: once stored over the fire of high As-content coal. x-axis: length of rice grain, y-axis: width of rice grain B: not baked over fire. Note: Quite different As concentration detected (A: range 500-2500, B: range 4-12)

The arsenic micro-distributions in rice grains collected in the hyper-endemic village (A) and control sites (B) in the same county are displayed in Figure 4(Fig. 4). It can be seen that an unexpected high density of the element arsenic was easily found in the husk of the rice grains, which were baked over fire in the hyper-endemic village. Surprisingly, a very high density of As was also shown in grain endosperm, the major edible part. However, As was not found at a detectable level in the embryo. Please notice that the scales at the y-axis for both mapping spectra are not identical. In contrast, the element As was hardly detected in any parts of the control samples, which were collected from a non-arseniasis area in the same county which was never exposed to any kind of As pollution. Similar pictures were also obtained when μ-XRF mapping was applied on the corn samples from the same areas (the mapping spectra of corn samples are not shown).

## Discussion

This is the first report on a study applying synchrotron radiation techniques, previously applied by Finkelman and colleagues on high arsenic coals in this area (Finkelman et al., 1999[4]), on food and hair samples on the rare case of endemic arseniasis related to indoor exposure to As. μ-XRF and XAFS are non-destructive physical approaches which need no chemical preparation procedures prior to the assay. The non-destructive,* in situ* assays provided more new, stimulating data which cannot be gained by any other conventional, neither chemical nor biochemical technique. The present approach will also presumably promote the investigation of other population health problems triggered by biogeochemical abnormality, such as endemic thallotoxicosis (Xiao et al., 2004[19]) or mercury poisoning (Feng and Qiu, 2008[3]) observed in the Southwest Guizhou area. 

Usually, arsenic levels in hair, especially the hair in females, can serve as a biomarker for short-term (<1 year) internal dose. In forensic medicine practice, variation of As levels in different sections of the same hair has been successfully used to recapitulate the chronic poisoning process and the approximate dosage applied. An example is the scientific deduction of possible historical events that happened in St Helena island during 1815-1821 when Napoléon Bonaparte was assumed to be gradually murdered by deliberate arsenic poisoning (Weider and Fournier, 1999[17]). In our case the As levels along the hair samples were found largely homogenous. It might be suggested that the exposure environment in this endemic village was, basically, stable around the time of sampling, so that any part of the hair samples would be suitable for the assay.

In our preliminary work in cooperation with SSRF, the X-ray fluorescence spectra of hair samples from the same endemic village suggested that there were no noticeable As peak area differences between two different groups of the residents (patients and their asymptomatic neighbors, preliminary data not shown). Furthermore, ICP-MS assays have been performed with all human hair samples collected, resulting in almost identical results for both groups of residents (F=0.001, P=0.981). This is totally different from observations reported so far from the arseniasis endemic areas related to As contaminated drinking water sources in this country and along the world as well. For final excluding of any possible interfering factors, we carefully checked the decades-long medical surveillance records archived in the Prefecture Centre for Disease Control (CDC) and made quite clear that the last medical dearsenization intervention with dimercaprol in this endemic population was conducted in 1990-1991 and covered only about 500 local people, only a small portion of the local population. Based on all the information available, it is reasonable to assume that the dearsenization intervention in part of the local residents in the early 1990s does not have a long-lasting impact on the As body burden in this exposed population.

The “hypernormal” phenomena observed in this investigation is well consistent with our previous observation. In our early work conducted in 2000 and in 2004 in the same population (Lin et al., 2003[8], 2006[10], 2010[12]), we found that As internal dosage expressed as total As level in hair and in urine in the subgroup of diagnosed arseniasis patients was not higher than in the subgroup of asymptomatic neighbors. The internal As dosage of ethnic Hmong subjects was found significantly higher than of their ethnic Han neighbors (p < 0.001 for As in hair and p < 0.01 for urine samples), though the arseniasis prevalence in Hmong farmers was significantly less profound than among the Han farmers (5.9 % *vs.* 32.7 %, OR: 0.12, 95 % CI: 0.06-0.27, P=3 x 10^-10^) (Lin et al., 2006[10]). 

Quite similar As levels in the hair samples collected from a thallotoxicosis-endemic village which is only about 10 km away from our target arseniasis-endemic village were confirmed by our current work. The exposure route for thallium is via locally produced food (Xiao et al., 2004[18]). Furthermore, these authors stated that local food is also the source for elevated urinary levels of arsenic and mercury. Thus, the impact of mercury should not be ignored. However, to our best knowledge no data on the local coal is available - in contrast to coals of the most eastern part of that province which is about 400 to 500 km from the investigated target village. However, chemical analysis of a coal sample used in another village (approximately 40 km from the investigated village) showed a mercury concentration of 55 ppm (Finkelman et al., 1999[4]). Interestingly, no typical arseniasis symptoms have ever been diagnosed in addition to the thallotoxicosis among the residents so far. All of these observations might suggest that the exposure to high level As in the environment or high As internal dosage is not inevitably related to an increased prevalence of arsenic-related skin symptoms. 

The X-ray absorption fine structure (XAFS) analysis revealed the coexistence of both the As^+3^ and As^+5^ states in every hair sample tested. However, the ratio of As^+3^ / As^+5^ varied greatly from person to person. Generally, the diagnosed patients displayed a clear tendency of higher As^+3^ / As^+5^ ratios than the asymptomatic fellow villagers. This finding is in line with the basic concept of toxicological science that As^+3^ is more toxic than As^+5^. 

A variance in the individual susceptibility to chronic arsenic poisoning has been suggested by Vahter (2000[16]). A remarkable clan aggregation was found in our preliminary survey in this village in April 2002 (Lin et al., 2003[8]). In a field investigation conducted in 2004, the ethnicity-dependent (or clan-dependent) variation in arseniasis prevalence was confirmed in this village, though all the families had been living together in the same village for generations and were exposed to similar indoor combustion of local high As coal for a quite similar time duration (Lin et al., 2006[10]). A few of the host factors, such as the polymorphisms at several loci of the genome were proved to be associated with the modulation of arseniasis risk in this exceptionally exposed rural population (Lin et al., 2006[10], 2007[11], 2010[12][9]). Recent data might add more power to support the pivot role of host factors.

For decades, the villagers in the endemic area have been taught persistently by local medical personnel and by local government officials that the grain or the vegetable must be water washed or rinsed thoroughly to remove the As before it can be cooked or ingested. The μ-XRF mapping displayed, for the first time, that the arsenic pollution of rice grains is not only restricted to the surface but has also penetrated deeply inside the grain and cumulated in the embryo of the grain, which is the major part of daily nutrition at a level which cannot be ignored any longer. Only a limited number of grain samples have been mapped by μ-XRF technique since the machine time of SSRF for the present study was limited. Therefore, our observation can only be considered as “preliminary”. More work is required. Our new data of micro-distribution of As deeply inside the grain may explain why the major item of the teaching program to the local residents, i.e. rinsing the grain with water several times, is largely ineffective. The results of this study should promote studies to investigate, whether rinsing with water remains also largely ineffective or not when applied to chili or corn, food which is very important for the arsenic load of the villagers in this area.

It must be mentioned that exposure conditions in this village have been improved in the last decades or so, since a series of countermeasures has been put forward in the endemic area by local authorities and by some other agencies as we have seen in our field investigation in April 2004 (Lin et al., 2006[10], 2010[12]). Our data of 2004 also witnessed a significant decrease of internal dose of total arsenic in the villagers. The hair As content in each subject obtained in the present study does no longer represent the real internal As dose of the subjects at the time of skin lesion onset or at the time of diagnosis. The 1991 survey, the first and the only overall field investigation ever held in the whole endemic area, including the target village of the present investigation was targeted only towards different exposure environments, not focused on specific individuals or subject subgroups. The retrospective assessment of As internal load in each individual remains impossible.

## Conclusion

Synchrotron radiation techniques, such as micro-beam X-ray fluorescence (μ-XRF) and X-ray absorption fine structure (XAFS), are suitable techniques to determine arsenic species concentrations in different parts of hair and rice grain samples. When rice is stored over the open fire of high As-content coal for drying, arsenic penetrates the endosperm, the major edible part of the grain. Thus rinsing the grain with water several times will remain largely ineffective.

## Conflict of interest

The authors declare no conflict of interest.

## Acknowledgements

The data of the present study were obtained at the BL15U and BL14W1 beamline stations of the Shanghai Synchrotron Radiation Facility (SSRF). The authors would like to thank the staff of both beamline stations for their assistance and valuable suggestions.

## Figures and Tables

**Table 1:**
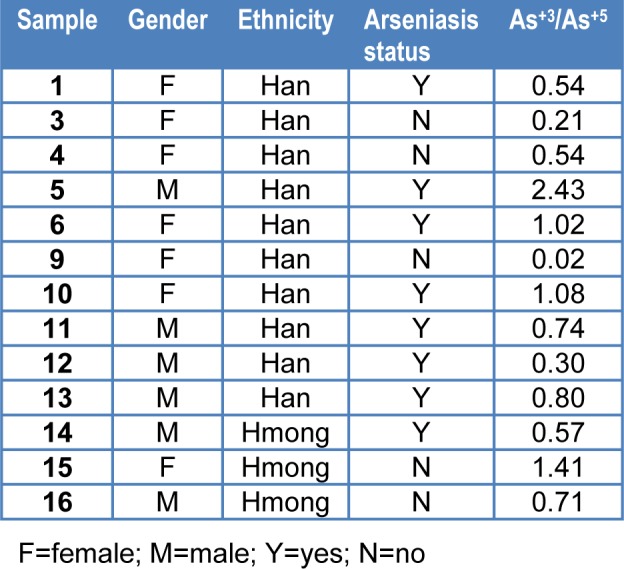
Ratio of As^+3^/As^+5^ in human hair samples from the arseniasis hyper-endemic village revealed by ICP-MS analysis

**Table 2:**
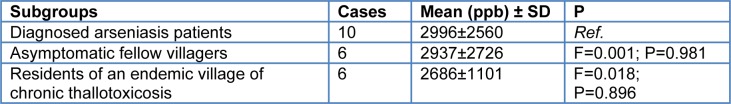
Comparison of As_75_ in hair samples of residents of an arseniasis-endemic village and of a thallotoxicosis-endemic village
